# Supplementary material for: Genome-Wide Identification, Evolution and Expression of the Complete Set of Cytoplasmic Ribosomal Protein Genes in Nile Tilapia
Source: Int J Mol Sci. 2020 Feb 12;21(4):1230. doi: 10.3390/ijms21041230 (PMC7072992; doi:10.3390/ijms21041230)
Supplement: Supplementary file 1 [file ijms-21-01230-s001.zip › Supplementary files/Supplementary Figures.docx]

**Supplementary Figures**

**
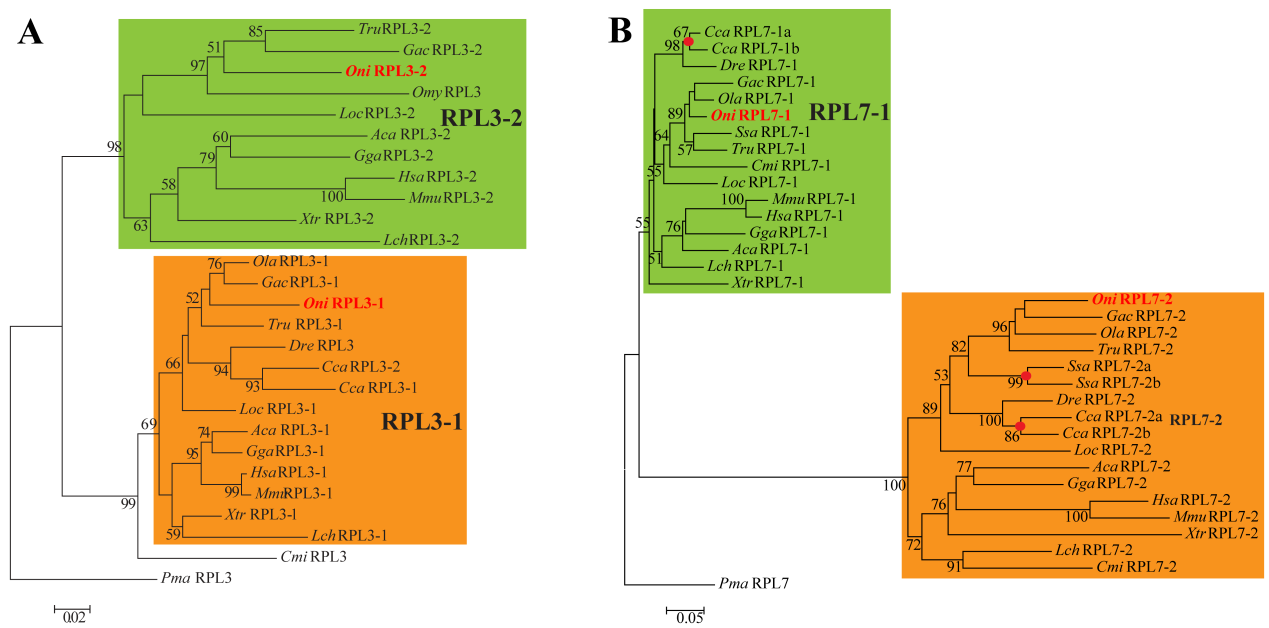

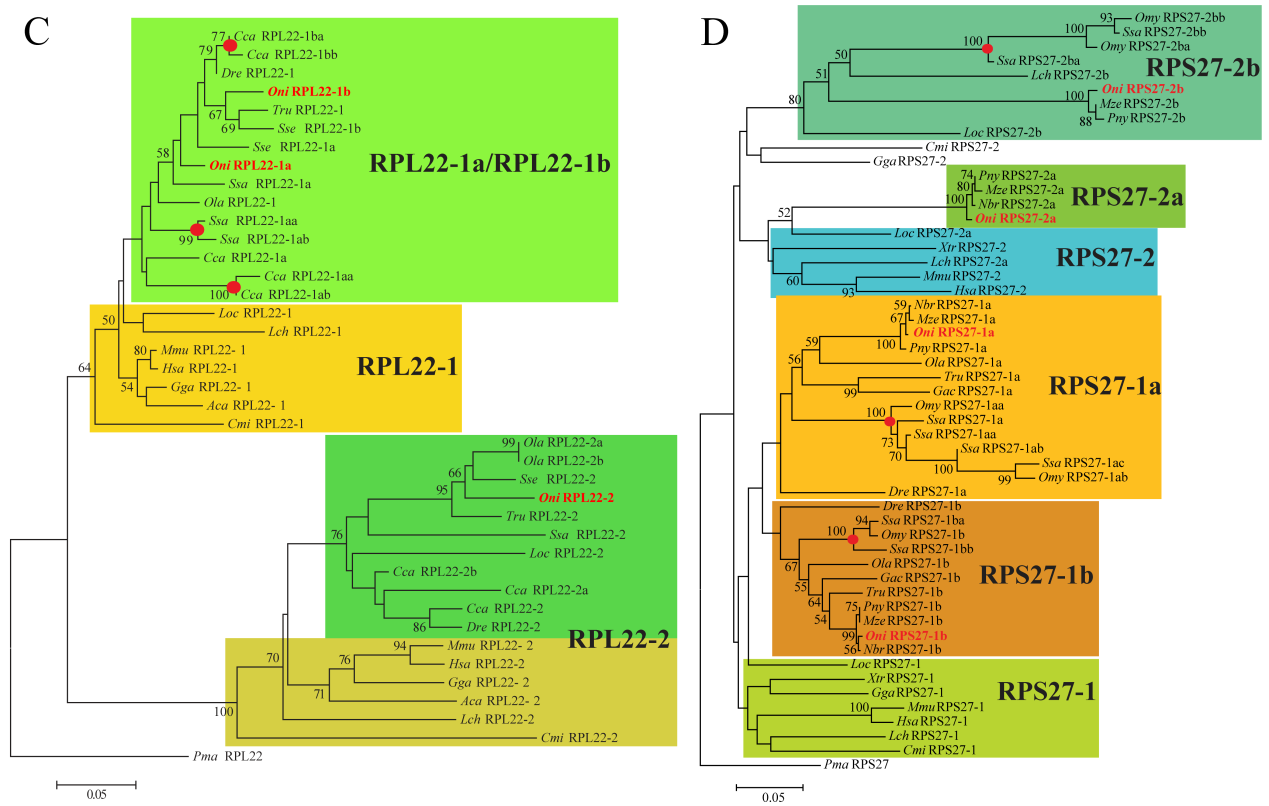

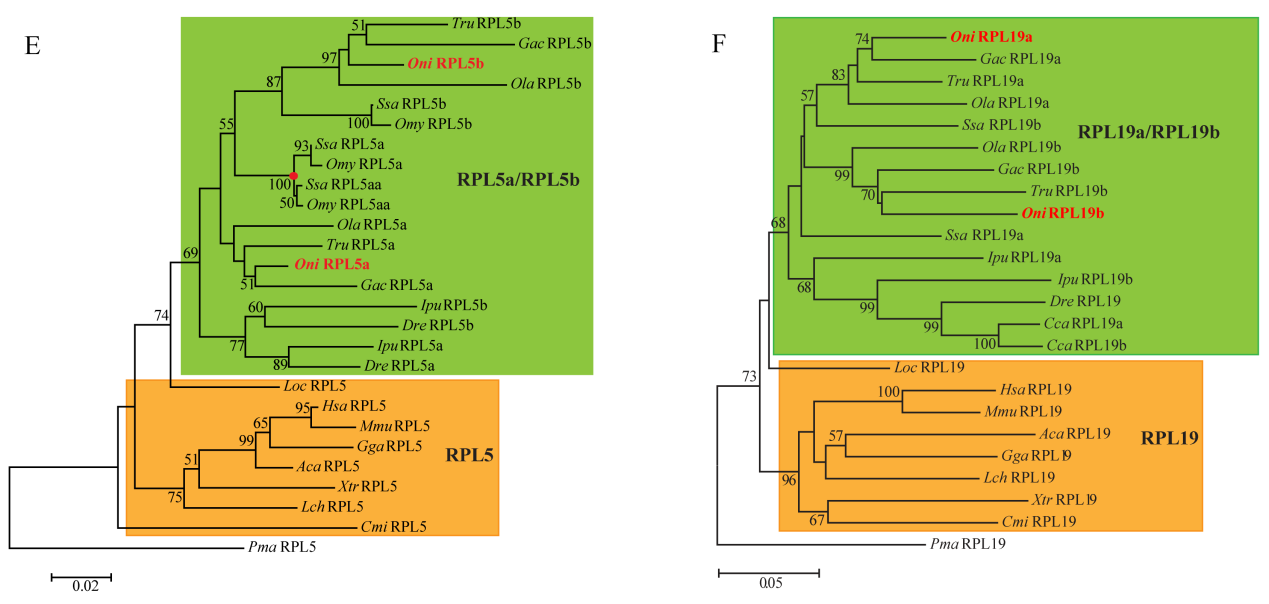

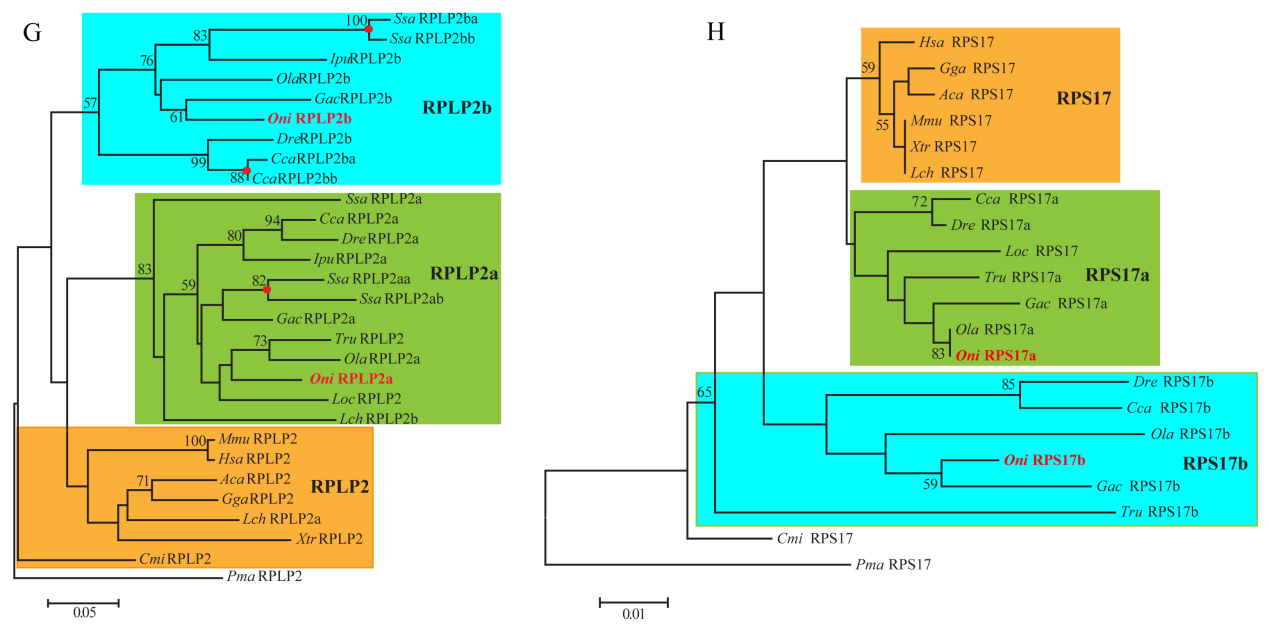

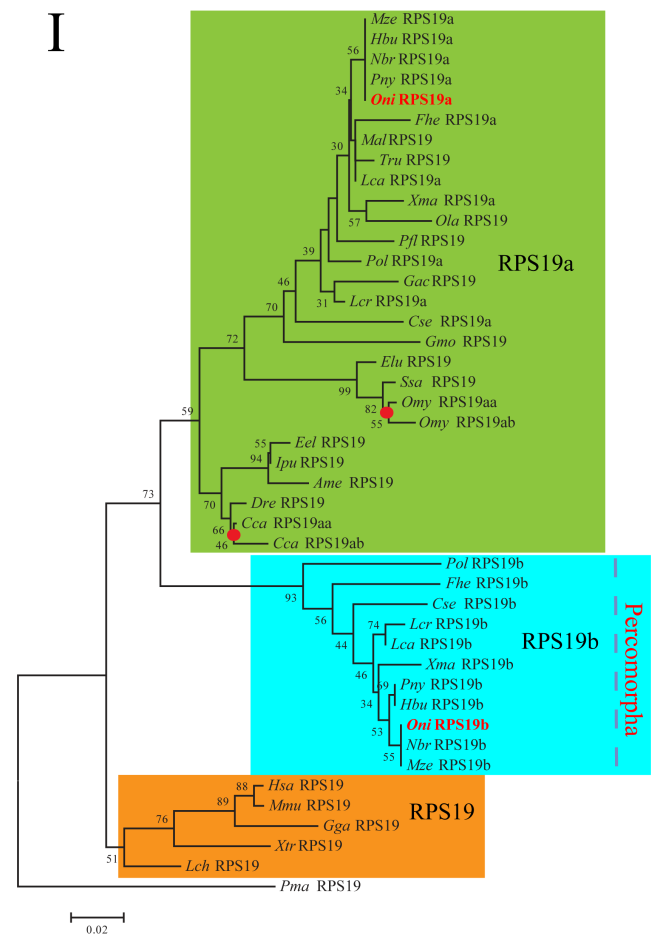
**

**Figure S1.** Phylogenetic tree of RPL3 (A), RPL7 (B), RPL22 (C), RPS27 (D), RPL5 (E), RPL19 (F), RPLP2 (G), RPS17 (H) and RPS19 (I) from tilapia and other vertebrates. Lamprey (*Petromyzon marinus*) was used as the outgroup to root each tree. Bootstrap values are indicated on each branch. Red dot indicates 4R event in some teleosts. The scale for branch length is shown below each tree. In the RP gene name, -1 and -2 indicate paralogs from 2R, a and b indicate paralogs from 3R, and aa, ab, ba and bb indicate paralogs from 4R. Different colors were used to indicate different clades. *Pma*, *Petromyzon marinus*; *Cmi*, *Callorhinchus milii*; *Lch*, *Latimeria chalumnae*; *Loc*, *Lepisosteus oculatus*; *Oni*, *Oreochromis niloticus*; *Tru*, *Takifugu rubripes*; *Dre*, *Danio rerio*; *Cca*, *Cyprinus carpio*; *Ipu*, *Ictalurus punctatus*; *Ola*, *Oryzias latipes*; *Gac*, *Gasterosteus aculeatus*; *Ssa*, *Salmo salar*; *Pny*, *Pundamilia nyererei*; *Mze*, *Maylandia zebra*; *Nbr*, *Neolamprologus brichardi*; *Hbu*, *Haplochromis burtoni*; *Lcr*, *Larimichthys crocea*; *Xma*, *Xiphophorus maculatus*; *Elu*, *Esox lucius*; *Eel*, *Electrophorus electricus*; *Ame*, *Astyanax mexicanus*; *Omy*, *Oncorhynchus mykiss*; *Lca*, *Lates calcarifer*; *Cse*, *Cynoglossus semilaevis*; *Pol*, *Paralichthys olivaceus*; *Fhe*, *Fundulus heteroclitus*; *Xtr*, *Xenopus tropicalis*; *Aca*, *Anolis carolinensis*; *Gga*, *Gallus gallus*; *Mmu*, *Mus musculus*; *Hsa*, *Homo sapiens*.


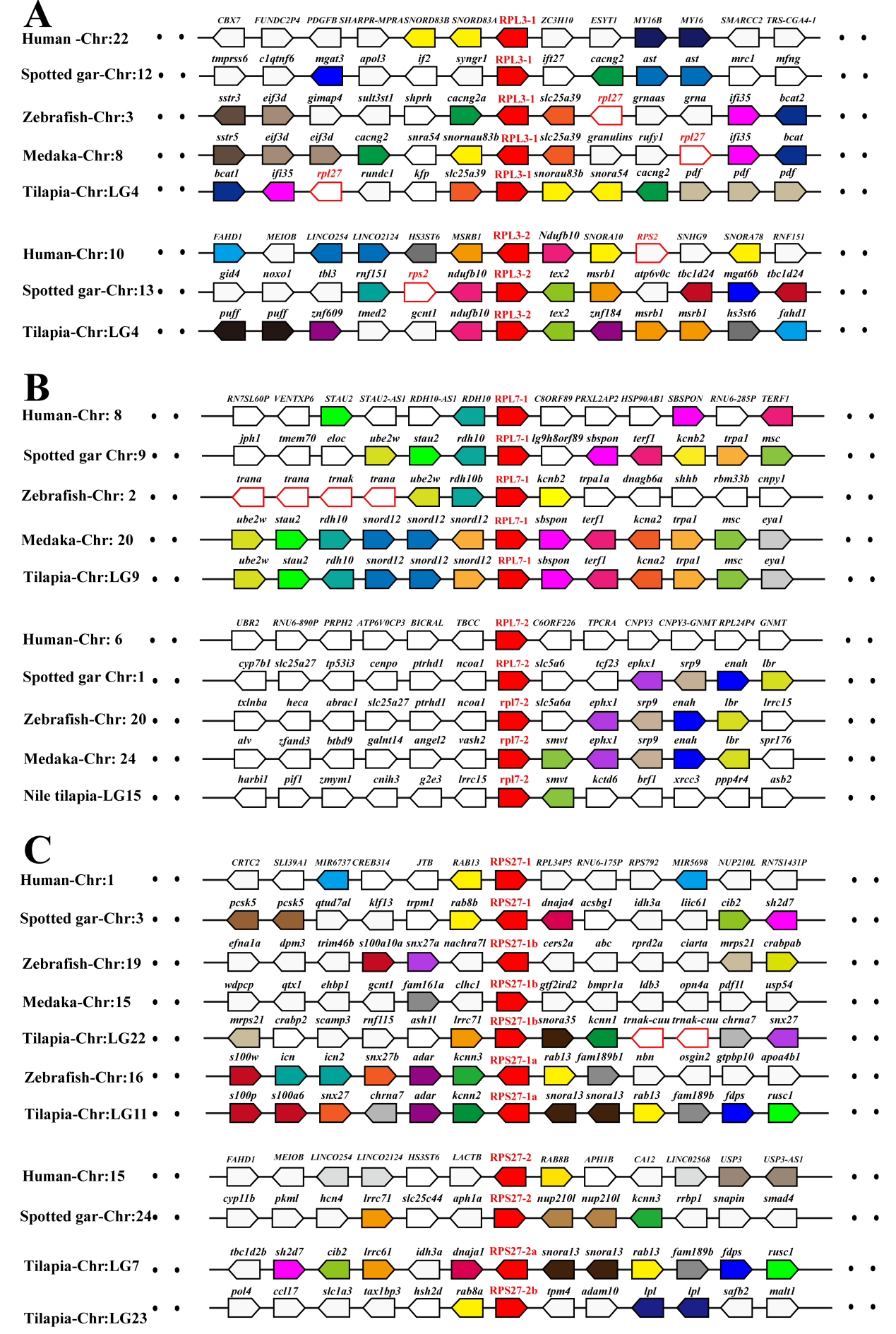

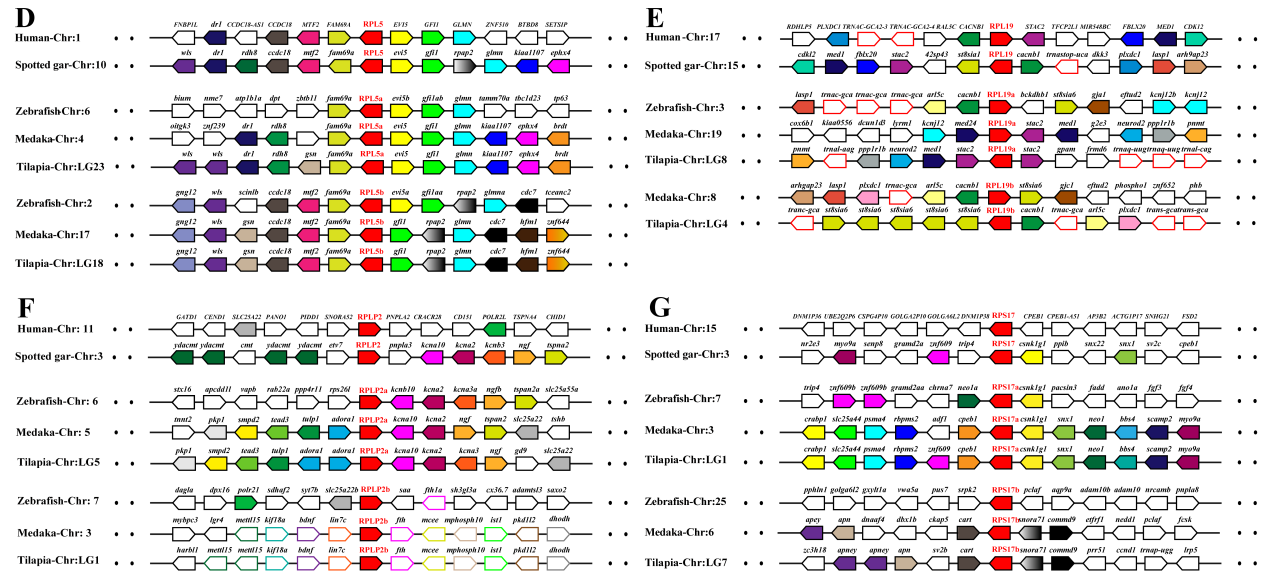


**Figure S2.** Synteny analyses of RPL3 (A), RPL7 (B), RPS27 (C), RPL5 (D), RPL19 (E), RPLP2 (F) and RPS17 (G) and their adjacent genes in tilapia and other vertebrates. Rectangles represent genes in chromosome/scaffold. Dotted lines represent omitted genes of the chromosome/scaffold. The direction of the arrows indicates the gene orientation. The RP genes are shown in red, while the other genes are shown in different color.
