# Supplementary material for: Genome-Wide Identification, Evolution and Expression of the Complete Set of Cytoplasmic Ribosomal Protein Genes in Nile Tilapia
Source: Int J Mol Sci. 2020 Feb 12;21(4):1230. doi: 10.3390/ijms21041230 (PMC7072992; doi:10.3390/ijms21041230)
Supplement: Supplementary file 1 [file ijms-21-01230-s001.zip › Supplementary files/Supplementary Figures.pdf]

Supplementary Figures

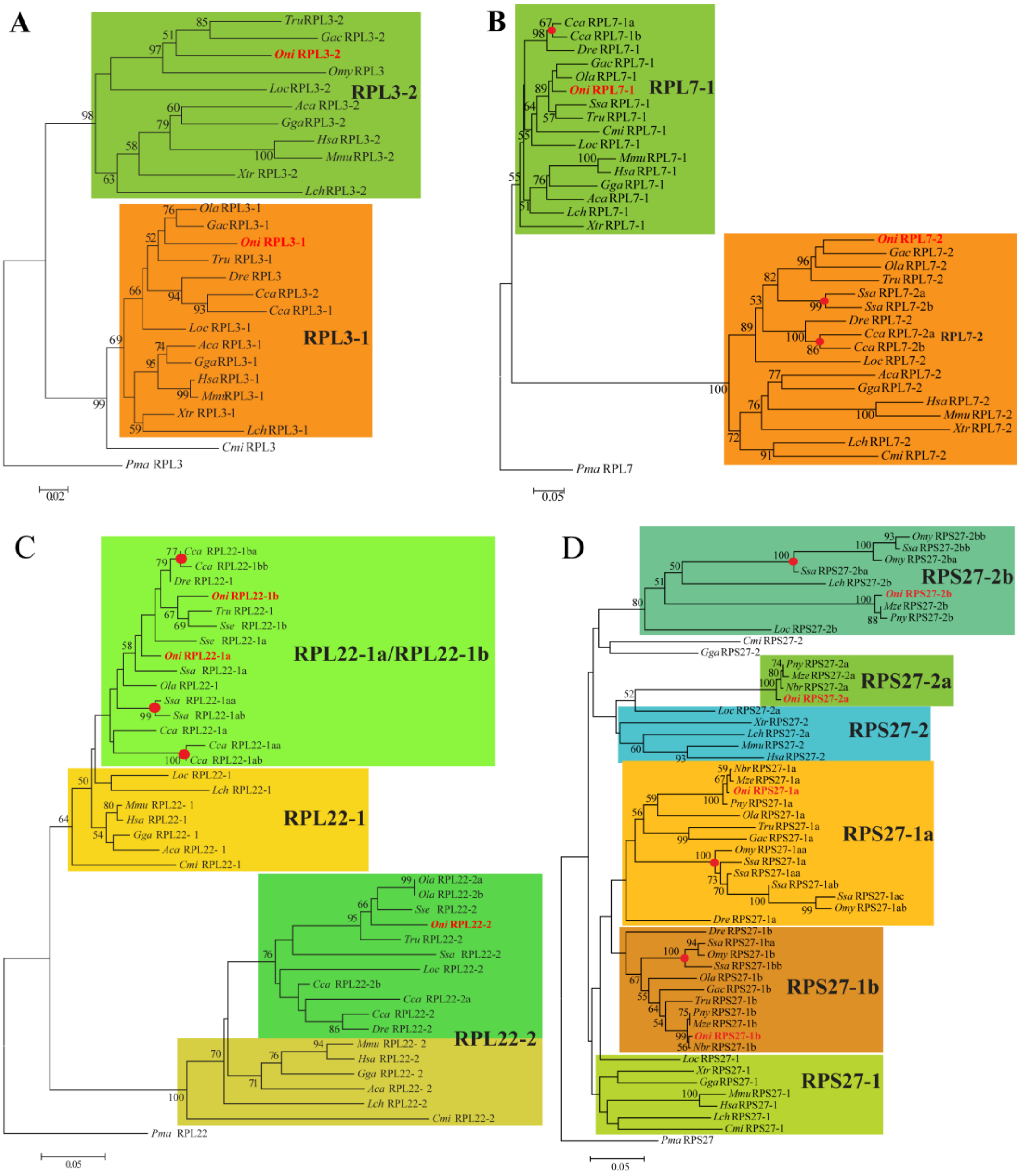

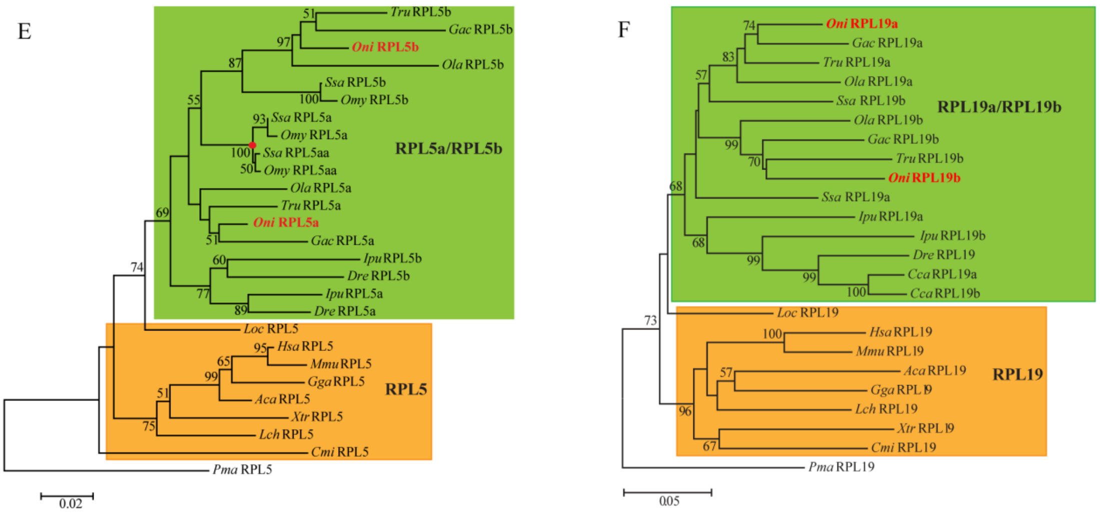
E
Tru RPL5b
Gac RPL5b
Oni RPL5b
Ola RPL5b
Ssa RPL5b
Omy RPL5b
Ssa RPL5a
Omy RPL5a
Ssa RPL5aa
Omy RPL5aa
Ola RPL5a
Tru RPL5a
Oni RPL5a
Gac RPL5a
Ipu RPL5b
Dre RPL5b
Ipu RPL5a
Dre RPL5a
RPL5a/RPL5b
Loc RPL5
Hsa RPL5
Mmu RPL5
Gga RPL5
Aca RPL5
Xtr RPL5
Lch RPL5
Cmi RPL5
RPL5
Pma RPL5
0.02
F
Oni RPL19a
Gac RPL19a
Tru RPL19a
Ola RPL19a
Ssa RPL19b
Ola RPL19b
Gac RPL19b
Tru RPL19b
Oni RPL19b
Ssa RPL19a
Ipu RPL19a
Ipu RPL19b
Dre RPL19
Cca RPL19a
Cca RPL19b
RPL19a/RPL19b
Loc RPL19
Hsa RPL19
Mmu RPL19
Aca RPL19
Gga RPL19
Lch RPL19
Xtr RPL19
Cmi RPL19
RPL19
Pma RPL19
0.05
4

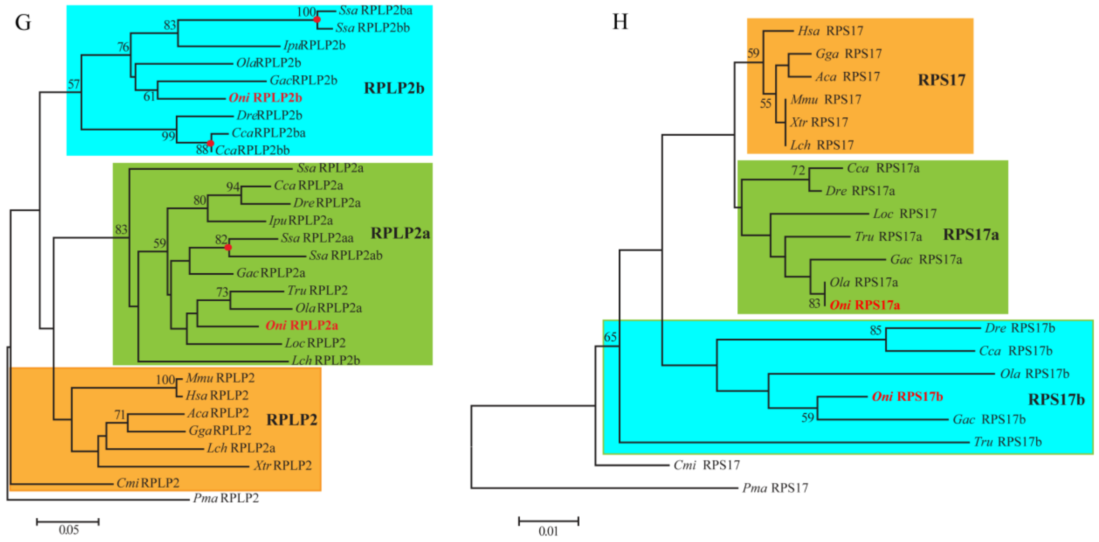
G
Ssa RPLP2ba
Ssa RPLP2bb
Ipu RPLP2b
Ola RPLP2b
Gac RPLP2b
Oni RPLP2b
Dre RPLP2b
Cca RPLP2ba
Cca RPLP2bb
RPLP2b
Ssa RPLP2a
Cca RPLP2a
Dre RPLP2a
Ipu RPLP2a
Ssa RPLP2aa
Ssa RPLP2ab
Gac RPLP2a
Tru RPLP2
Ola RPLP2a
Oni RPLP2a
Loc RPLP2
Lch RPLP2b
RPLP2a
Mmu RPLP2
Hsa RPLP2
Aca RPLP2
Gga RPLP2
Lch RPLP2a
Xtr RPLP2
Cmi RPLP2
RPLP2
Pma RPLP2
0.05
H
Hsa RPS17
Gga RPS17
Aca RPS17
Mmu RPS17
Xtr RPS17
Lch RPS17
RPS17
Cca RPS17a
Dre RPS17a
Loc RPS17
Tru RPS17a
Gac RPS17a
Ola RPS17a
Oni RPS17a
RPS17a
Dre RPS17b
Cca RPS17b
Ola RPS17b
Oni RPS17b
Gac RPS17b
Tru RPS17b
RPS17b
Cmi RPS17
Pma RPS17
0.01
5

I

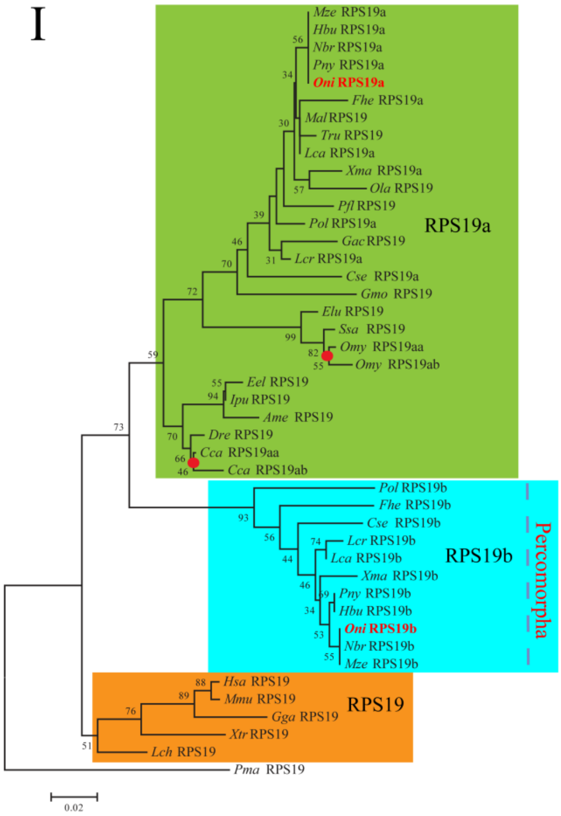

Figure S1. Phylogenetic tree of RPL3 (A), RPL7 (B), RPL22 (C), RPS27 (D), RPL5 (E), RPL19 (F), RPLP2 (G), RPS17 (H) and RPS19 (I) from tilapia and other vertebrates. Lamprey (*Petromyzon marinus*) was used as the outgroup to root each tree. Bootstrap values are indicated on each branch. Red dot indicates 4R event in some teleosts. The scale for branch length is shown below each tree. In the RP gene name, -1 and -2 indicate paralogs from 2R, a and b indicate paralogs from 3R, and aa, ab, ba and bb indicate paralogs from 4R. Different colors were used to indicate different clades. *Pma, Petromyzon marinus*; *Cmi, Callorhinchus milii*; *Lch, Latimeria chalumnae*; *Loc, Lepisosteus oculatus*; *Oni, Oreochromis niloticus*; *Tru, Takifugu rubripes*; *Dre, Danio rerio*; *Cca, Cyprinus carpio*; *Ipu, Ictalurus punctatus*; *Ola, Oryzias latipes*; *Gac, Gasterosteus aculeatus*; *Ssa, Salmo salar*; *Pny, Pundamilia nyererei*; *Mze, Maylandia zebra*; *Nbr, Neolamprologus brichardi*; *Hbu, Haplochromis burtoni*; *Lcr, Larimichthys crocea*; *Xma, Xiphophorus maculatus*; *Elu, Esox lucius*; *Eel, Electrophorus electricus*; *Ame, Astyanax mexicanus*; *Omy, Oncorhynchus mykiss*; *Lca, Lates calcarifer*; *Cse, Cynoglossus semilaevis*; *Pol, Paralichthys olivaceus*; *Fhe, Fundulus heteroclitus*; *Xtr, Xenopus tropicalis*; *Aca, Anolis carolinensis*; *Gga, Gallus gallus*; *Mmu, Mus musculus*; *Hsa, Homo sapiens*.

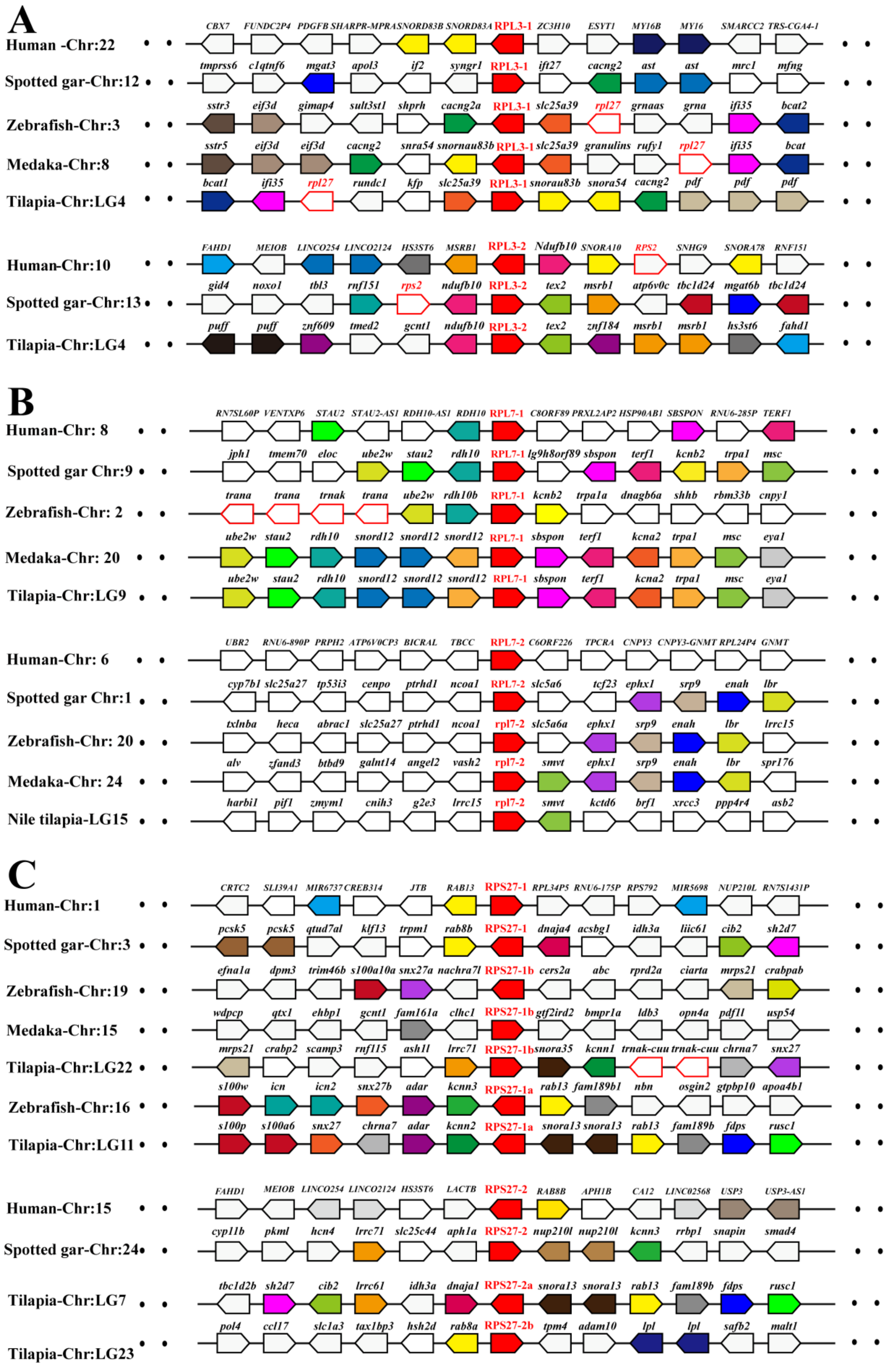
A
Human -Chr:22
CBX7 FUNDC2P4 PDGFB SHARPR-MPRASNORD83B SNORD83A RPL3-1 ZC3H10 ESYT1 MY16B MY16 SMARCC2 TRS-CGA4-1
Spotted gar-Chr:12
tmprss6 c1qtnf6 mgat3 apol3 if2 syngr1 RPL3-1 ift27 cacng2 ast ast mrc1 mfng
Zebrafish-Chr:3
sstr3 eif3d gimap4 sult3st1 shprh cacng2a RPL3-1 slc25a39 rpl27 grnaas grna ifi35 bcat2
Medaka-Chr:8
sstr5 eif3d eif3d cacng2 snra54 snornau83b RPL3-1 slc25a39 granulins rufy1 rpl27 ifi35 bcat
Tilapia-Chr:LG4
bcat1 ifi35 rpl27 rundc1 kfp slc25a39 RPL3-1 snorau83b snora54 cacng2 pdf pdf pdf
Human-Chr:10
FAHD1 MEIOB LINCO254 LINCO2124 HS3ST6 MSRB1 RPL3-2 Ndufb10 SNORA10 RPS2 SNHG9 SNORA78 RNF151
Spotted gar-Chr:13
gid4 noxo1 tbl3 rnf151 rps2 ndufb10 RPL3-2 tex2 msrb1 atp6v0c tbc1d24 mgat6b tbc1d24
Tilapia-Chr:LG4
puff puff znf609 tmed2 gcnt1 ndufb10 RPL3-2 tex2 znf184 msrb1 msrb1 hs3st6 fahd1
B
Human-Chr: 8
RN7SL60P VENTXP6 STAU2 STAU2-AS1 RDH10-AS1 RDH10 RPL7-1 C8ORF89 PRXL2AP2 HSP90AB1 SBSPON RNU6-285P TERF1
Spotted gar Chr:9
jph1 tmem70 eloc ube2w stau2 rdh10 RPL7-1 lg9h8orf89 sbspon terf1 kcnb2 trpa1 msc
Zebrafish-Chr: 2
trana trana trnak trana ube2w rdh10b RPL7-1 kcnb2 trpa1a dnagb6a shhb rbm33b cnpy1
Medaka-Chr: 20
ube2w stau2 rdh10 snord12 snord12 snord12 RPL7-1 sbspon terf1 kcna2 trpa1 msc eya1
Tilapia-Chr:LG9
ube2w stau2 rdh10 snord12 snord12 snord12 RPL7-1 sbspon terf1 kcna2 trpa1 msc eya1
Human-Chr: 6
UBR2 RNU6-890P PRPH2 ATP6V0CP3 BICRAL TBCC RPL7-2 C6ORF226 TPCRA CNPY3 CNPY3-GNMT RPL24P4 GNMT
Spotted gar Chr:1
cyp7b1 slc25a27 tp53i3 cenpo ptrhd1 ncoa1 RPL7-2 slc5a6 tcf23 ephx1 srp9 enah lbr
Zebrafish-Chr: 20
txlnba heca abracl slc25a27 ptrhd1 ncoa1 rpl7-2 slc5a6a ephx1 srp9 enah lbr lrrc15
Medaka-Chr: 24
alv zfand3 btbd9 galnt14 angel2 vash2 rpl7-2 smvt ephx1 srp9 enah lbr spr176
Nile tilapia-LG15
harbi1 pif1 zmym1 cnih3 g2e3 lrrc15 rpl7-2 smvt kctd6 brf1 xrcc3 ppp4r4 asb2
C
Human-Chr:1
CRTC2 SLI39A1 MIR6737 CREB314 JTB RAB13 RPS27-1 RPL34P5 RNU6-175P RPS792 MIR5698 NUP210L RN7SL431P
Spotted gar-Chr:3
pcsk5 pcsk5 qtud7al klf13 trpm1 rab8b RPS27-1 dnaja4 acsbg1 idh3a liic61 cib2 sh2d7
Zebrafish-Chr:19
efna1a dpm3 trim46b s100a10a snx27a nachra7l RPS27-1b cers2a abc rprd2a ciarta mrps21 crabpab
Medaka-Chr:15
wdpcp qtx1 ehbp1 gcnt1 fam161a clhc1 RPS27-1b gtf2ird2 bmpr1a ldb3 opn4a pdf1l usp54
Tilapia-Chr:LG22
mrps21 crabp2 scamp3 rnf115 ash1l lrrc71 RPS27-1b snora35 kcnn1 trnak-cuu trnak-cuu chrna7 snx27
Zebrafish-Chr:16
s100w icn icn2 snx27b adar kcnn3 RPS27-1a rab13 fam189b1 nbn osgin2 gtpbp10 apoa4b1
Tilapia-Chr:LG11
s100p s100a6 snx27 chrna7 adar kcnn2 RPS27-1a snora13 snora13 rab13 fam189b fdps rusc1
Human-Chr:15
FAHD1 MEIOB LINCO254 LINCO2124 HS3ST6 LACTB RPS27-2 RAB8B APH1B CA12 LINC02568 USP3 USP3-AS1
Spotted gar-Chr:24
cyp11b pkml hcn4 lrrc71 slc25c44 aph1a RPS27-2 nup210l nup210l kcnn3 rrbp1 snapin smad4
Tilapia-Chr:LG7
tbc1d2b sh2d7 cib2 lrrc61 idh3a dnaja1 RPS27-2a snora13 snora13 rab13 fam189b fdps rusc1
Tilapia-Chr:LG23
pol4 ccl17 slc1a3 tax1bp3 hsh2d rab8a RPS27-2b tpm4 adam10 lpl lpl safb2 malt1

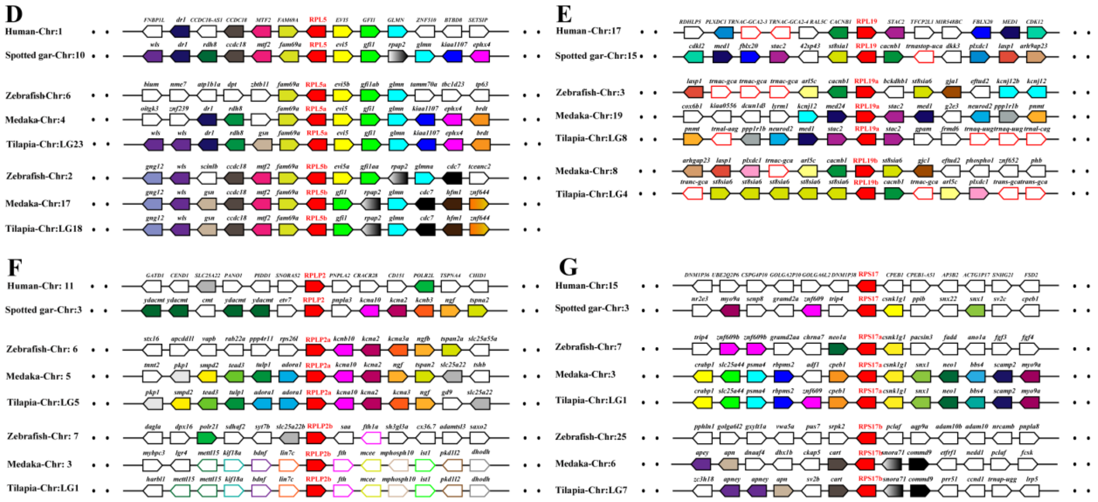

Figure S2. Synteny analyses of RPL3 (A), RPL7 (B), RPS27 (C), RPL5 (D), RPL19 (E), RPLP2 (F) and RPS17 (G) and their adjacent genes in tilapia and other vertebrates. Rectangles represent genes in chromosome/scaffold. Dotted lines represent omitted genes of the chromosome/scaffold. The direction of the arrows indicates the gene orientation. The RP genes are shown in red, while the other genes are shown in different color.
