# Supplementary figures and images for: Genome-Wide Identification, Evolution and Expression of the Complete Set of Cytoplasmic Ribosomal Protein Genes in Nile Tilapia
Source: Int J Mol Sci. 2020 Feb 12;21(4):1230. doi: 10.3390/ijms21041230 (PMC7072992; doi:10.3390/ijms21041230)

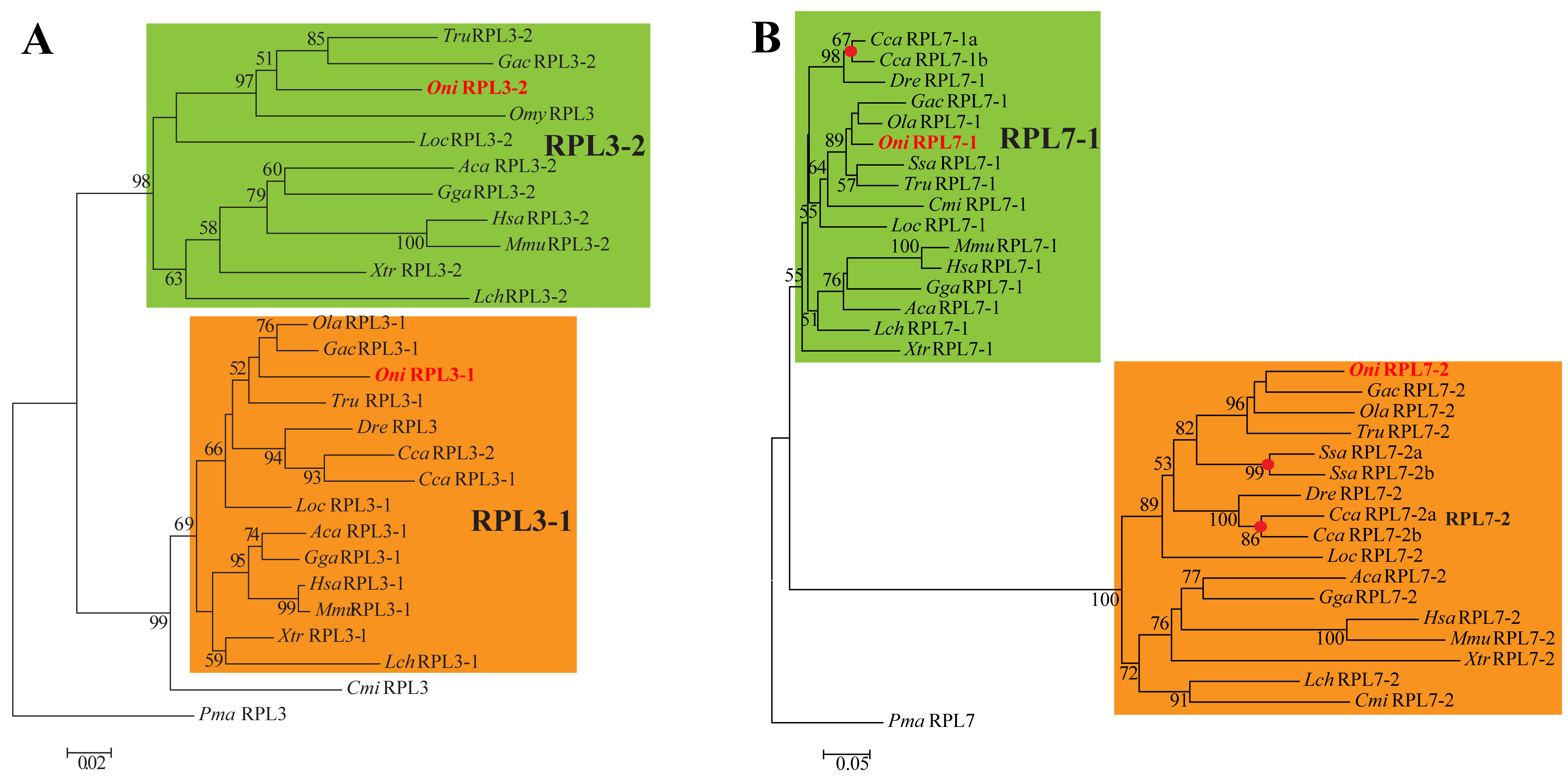

Supplement: Supplementary file 1 [file ijms-21-01230-s001.zip › Supplementary files/Supplementary Figure S1/Figure S1 A and B.tif]

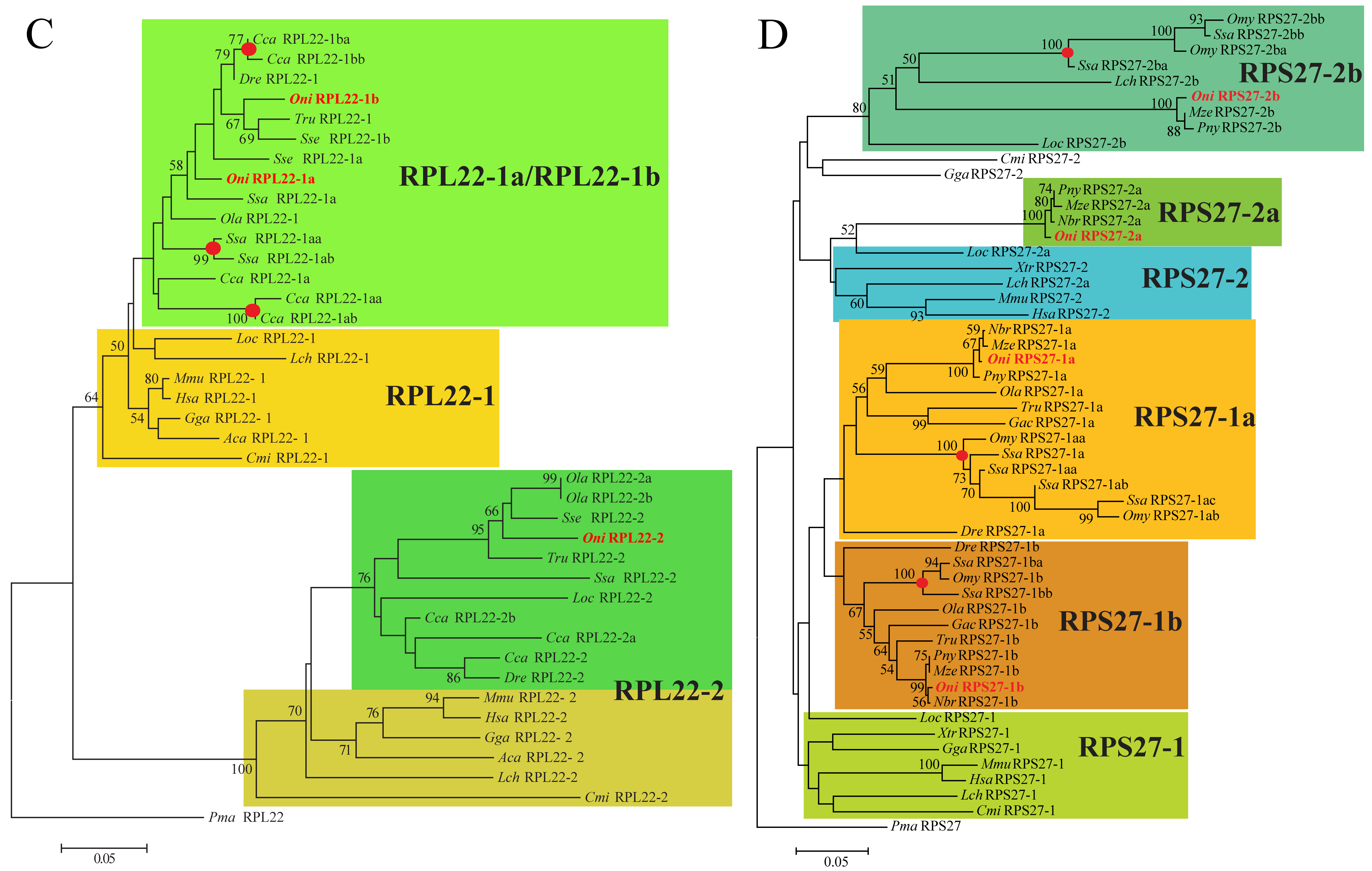

Supplement: Supplementary file 1 [file ijms-21-01230-s001.zip › Supplementary files/Supplementary Figure S1/Figure S1 C and D.tif]

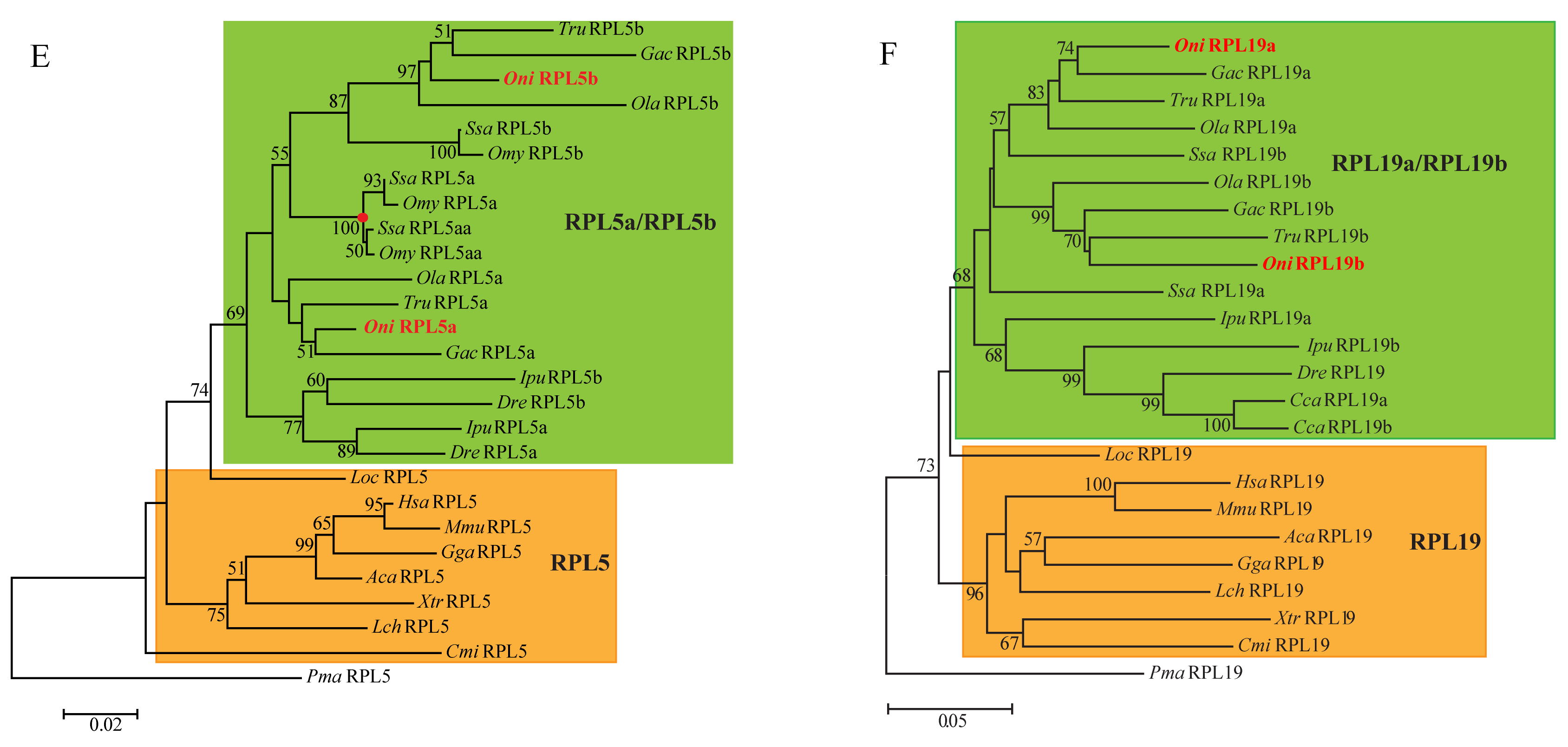

Supplement: Supplementary file 1 [file ijms-21-01230-s001.zip › Supplementary files/Supplementary Figure S1/Figure S1 E and F.tif]

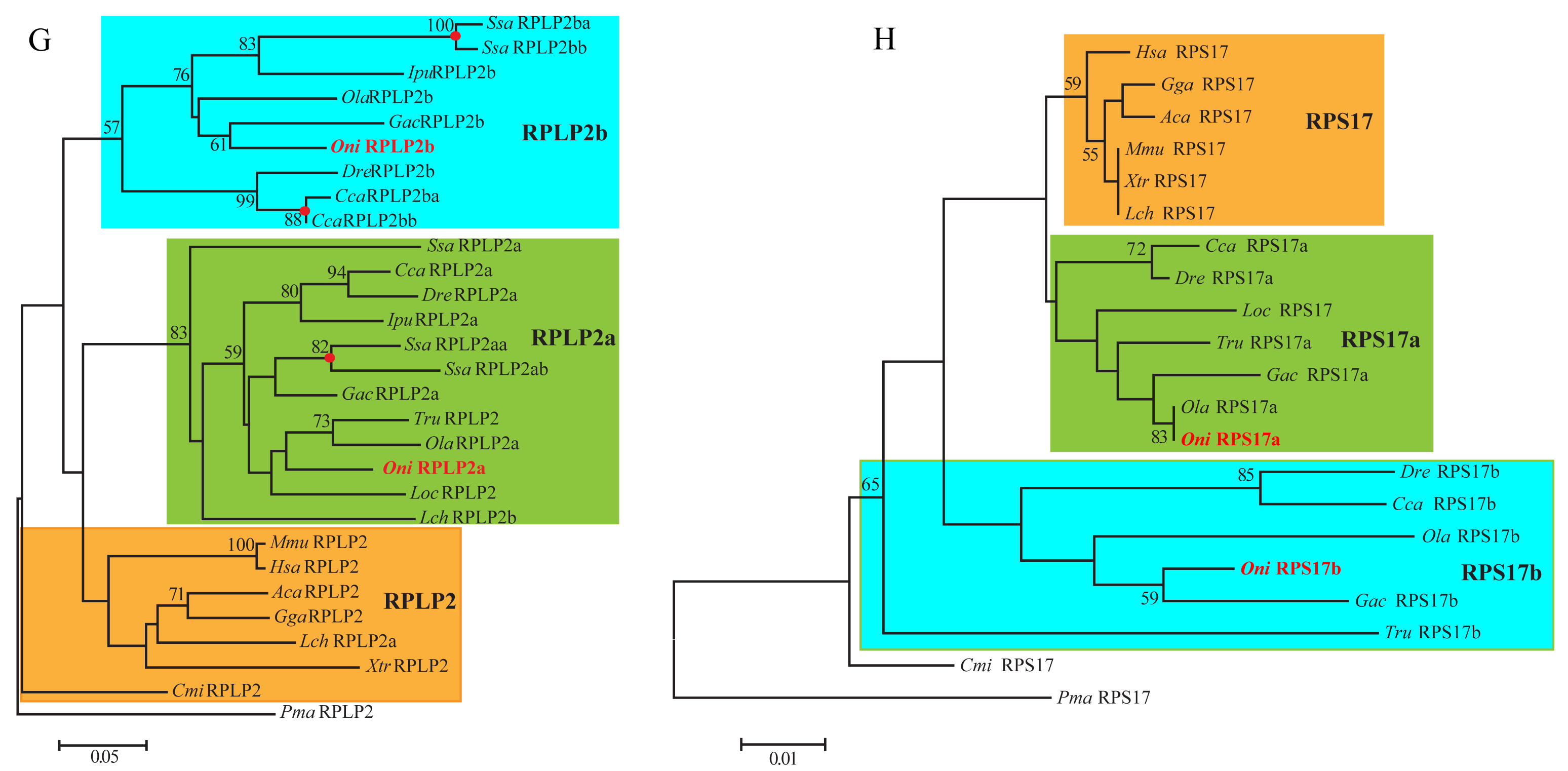

Supplement: Supplementary file 1 [file ijms-21-01230-s001.zip › Supplementary files/Supplementary Figure S1/Figure S1 G and H.tif]

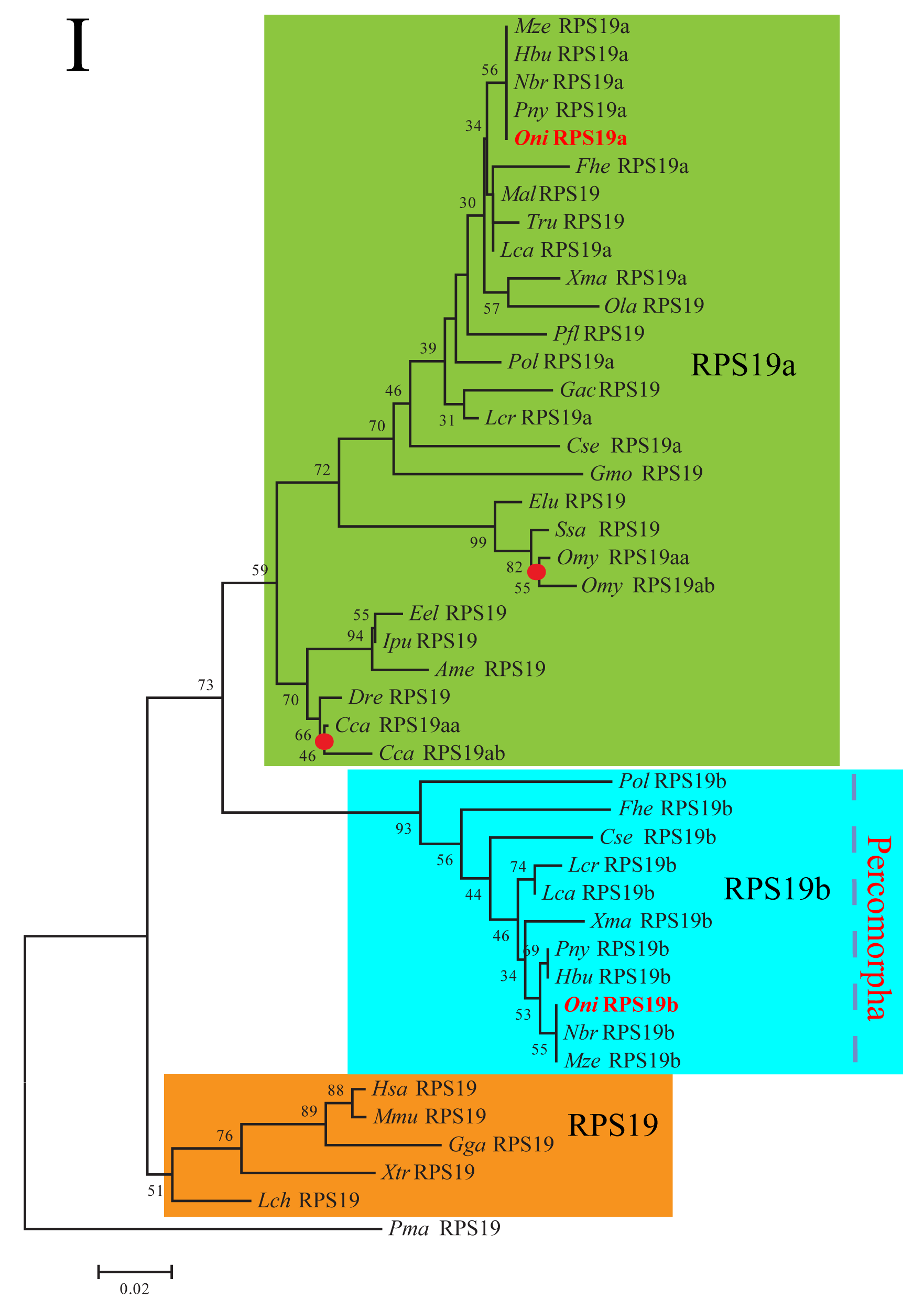

Supplement: Supplementary file 1 [file ijms-21-01230-s001.zip › Supplementary files/Supplementary Figure S1/Figure S1 I.tif]

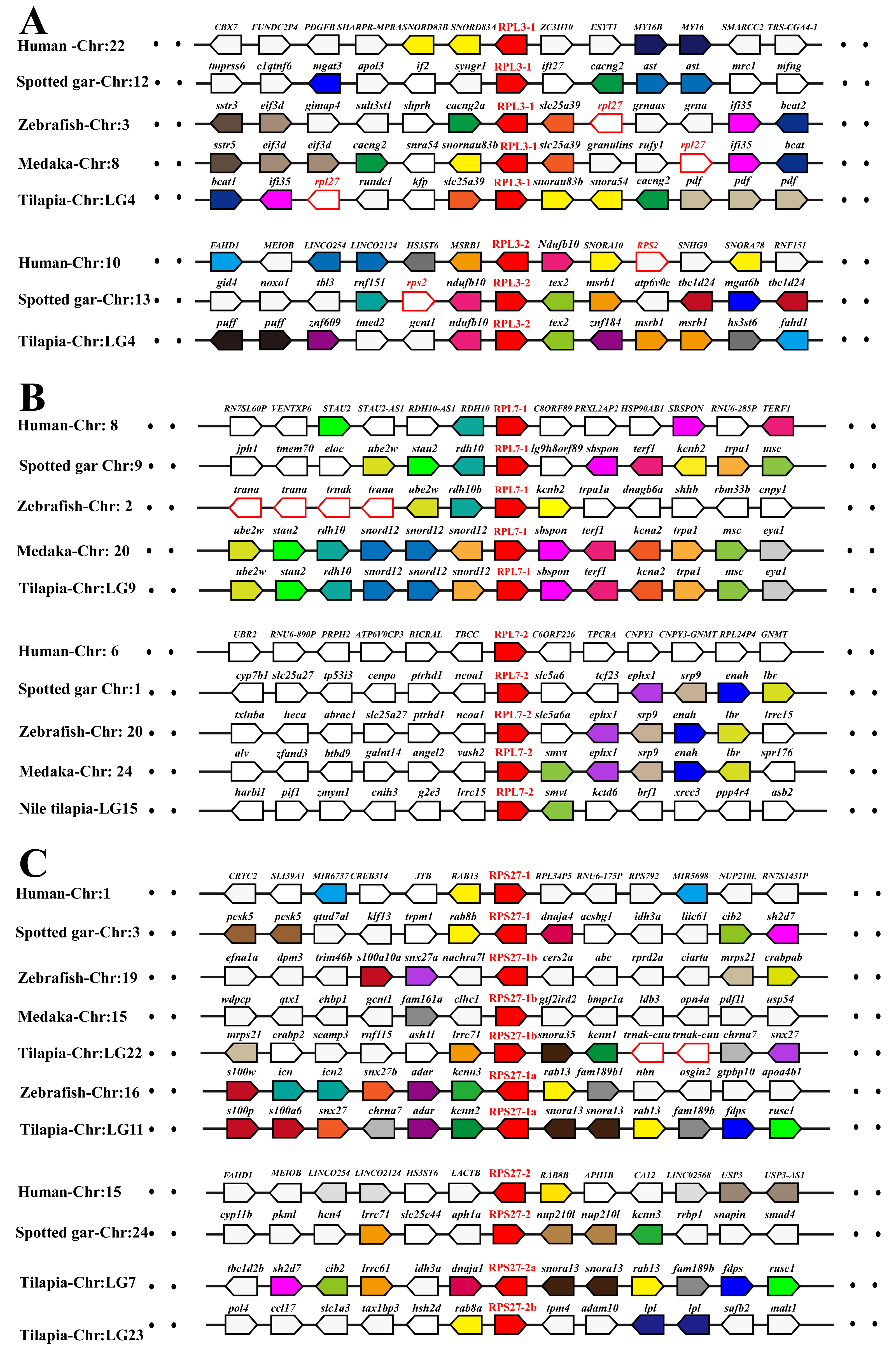

Supplement: Supplementary file 1 [file ijms-21-01230-s001.zip › Supplementary files/Supplementary Figure S2/20200122 Figure S2 synteny A,B,C.tif]

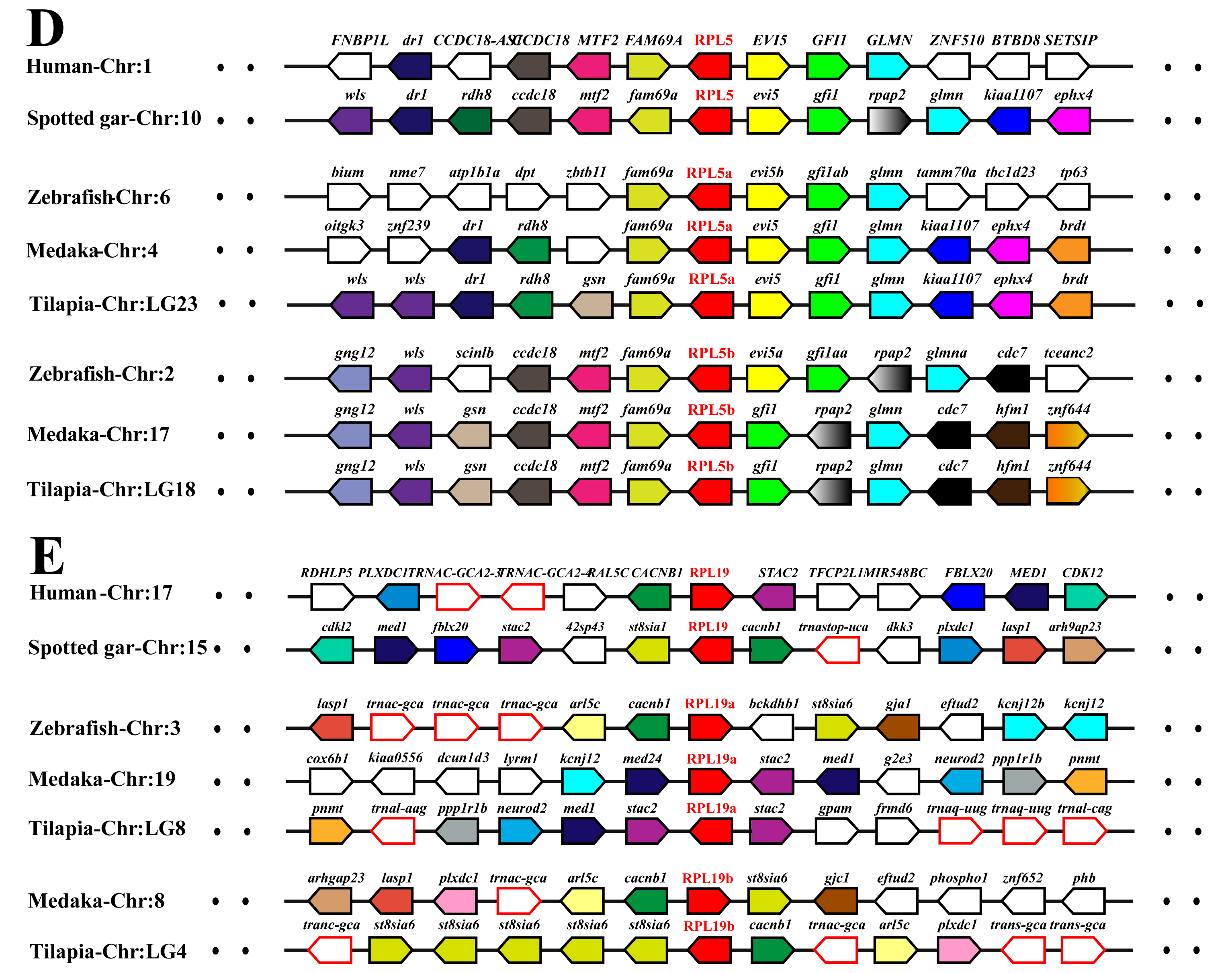

Supplement: Supplementary file 1 [file ijms-21-01230-s001.zip › Supplementary files/Supplementary Figure S2/20200122 Figure S2 synteny D,E.tif]

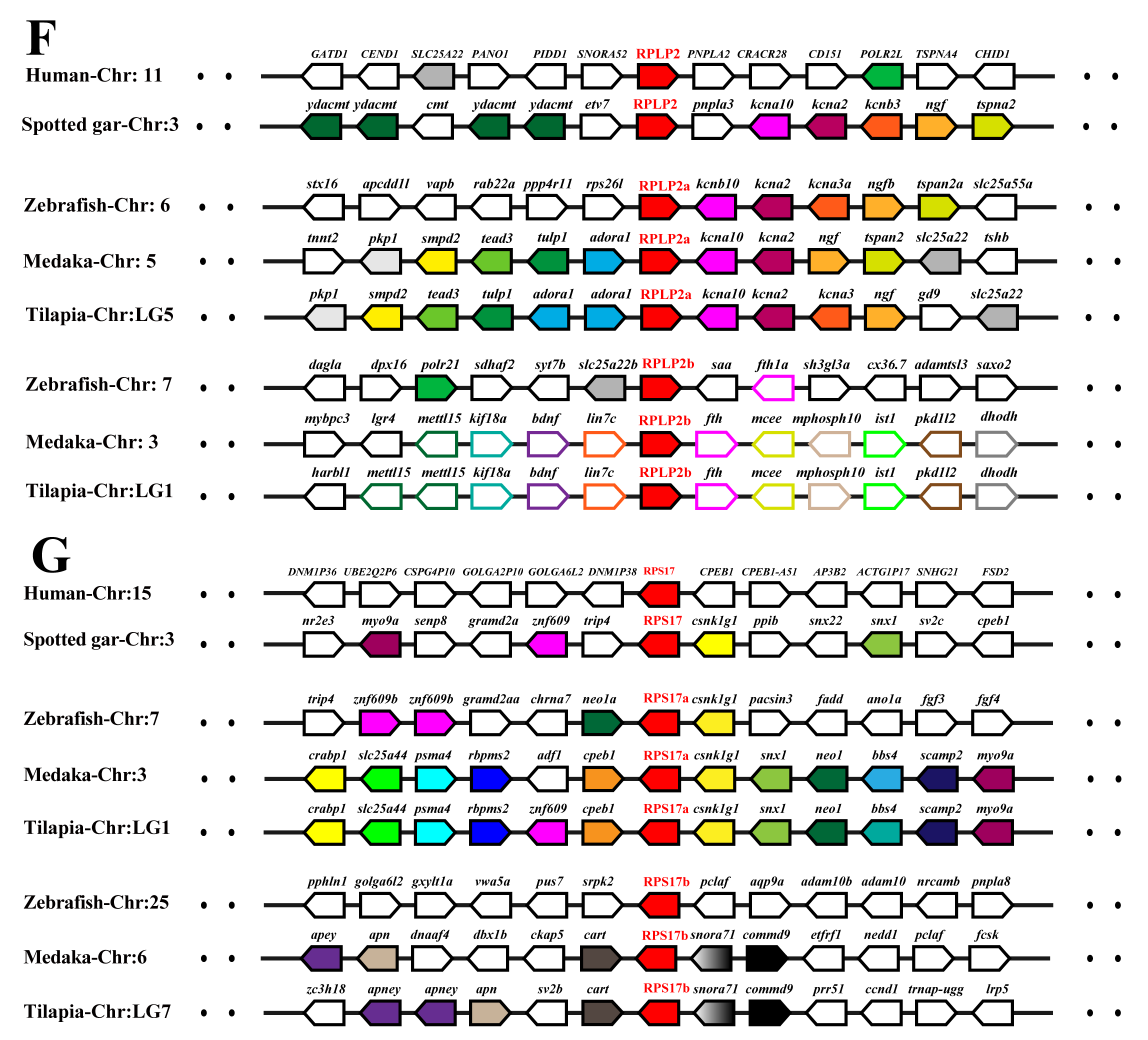

Supplement: Supplementary file 1 [file ijms-21-01230-s001.zip › Supplementary files/Supplementary Figure S2/20200122 Figure S2 synteny F,G.tif]
